# A Gamma-Knife-Enabled Mouse Model of Cerebral Single-Hemisphere Delayed Radiation Necrosis

**DOI:** 10.1371/journal.pone.0139596

**Published:** 2015-10-06

**Authors:** Xiaoyu Jiang, Liya Yuan, John A. Engelbach, Jeremy Cates, Carlos J. Perez-Torres, Feng Gao, Dinesh Thotala, Robert E. Drzymala, Robert E. Schmidt, Keith M. Rich, Dennis E. Hallahan, Joseph J. H. Ackerman, Joel R. Garbow

**Affiliations:** 1 Department of Chemistry, Washington University, St. Louis, Missouri, United States of America; 2 Department of Neurosurgery, Washington University, St. Louis, Missouri, United States of America; 3 Department of Radiology, Washington University, St. Louis, Missouri, United States of America; 4 Department of Radiation Oncology, Washington University, St. Louis, Missouri, United States of America; 5 Division of Biostatistics, Washington University, St. Louis, Missouri, United States of America; 6 Alvin J. Siteman Cancer Center, Washington University, St. Louis, Missouri, United States of America; 7 Department of Neuropathology, Washington University, St. Louis, Missouri, United States of America; 8 Department of Internal Medicine, Washington University, St. Louis, Missouri, United States of America; ENEA, ITALY

## Abstract

**Purpose:**

To develop a Gamma Knife-based mouse model of late time-to-onset, cerebral radiation necrosis (RN) with serial evaluation by magnetic resonance imaging (MRI) and histology.

**Methods and Materials:**

Mice were irradiated with the Leksell Gamma Knife^®^ (GK) Perfexion^TM^ (Elekta AB; Stockholm, Sweden) with total single-hemispheric radiation doses (TRD) of 45- to 60-Gy, delivered in one to three fractions. RN was measured using T2-weighted MR images, while confirmation of tissue damage was assessed histologically by hematoxylin & eosin, trichrome, and PTAH staining.

**Results:**

MRI measurements demonstrate that TRD is a more important determinant of both time-to-onset and progression of RN than fractionation. The development of RN is significantly slower in mice irradiated with 45-Gy than 50- or 60-Gy, where RN development is similar. Irradiated mouse brains demonstrate all of the pathologic features observed clinically in patients with confirmed RN. A semi-quantitative (0 to 3) histologic grading system, capturing both the extent and severity of injury, is described and illustrated. Tissue damage, as assessed by a histologic score, correlates well with total necrotic volume measured by MRI (correlation coefficient = 0.948, with p<0.0001), and with post-irradiation time (correlation coefficient = 0.508, with p<0.0001).

**Conclusions:**

Following GK irradiation, mice develop late time-to-onset cerebral RN histology mirroring clinical observations. MR imaging provides reliable quantification of the necrotic volume that correlates well with histologic score. This mouse model of RN will provide a platform for mechanism of action studies, the identification of imaging biomarkers of RN, and the development of clinical studies for improved mitigation and neuroprotection.

## Introduction

Despite recent advances in radiation treatment planning and delivery, onset of late-delayed radiation-induced neurotoxicity (radiation necrosis, RN) remains an important clinical problem characterized by debilitating clinical symptoms, a worsening quality of life, and even death. RN develops in up to 24% of patients receiving focal irradiation three or more months following therapy [1]. The incidence of RN is often higher with concurrent chemotherapy [2].

The lack of a well-developed, small-animal model of radiation necrosis has significantly hampered the development of diagnostic and therapeutic management of cerebral RN. Until recently, reports of small-animal models of radiation necrosis in brain tissue have been sparse, with most previous animal models developed in rats [3–8]. Several years ago, we described a murine model of radiation necrosis using a micro-radiotherapy (microRT) system [9]. The extent and progression of necrosis was characterized using contrast-enhanced T1- and T2-weighted MRI and the histology reflected changes typically seen in radiation necrosis in patients. Although our studies and those of others demonstrated the feasibility of generating radiation necrosis in mouse/rat brain using focal brain irradiation, they were all performed on small cohorts of animals. Determinants of the onset and progression of radiation necrosis, including radiation dose and fractionation, which are crucial for the design of studies aimed at developing methods to identify/detect, monitor, protect against, and mitigate radiation necrosis, have not been well investigated.

The Leksell Gamma Knife^®^ (GK) Perfexion^TM^, a state-of-the-art unit designed for stereotactic irradiation of patients with benign and malignant brain tumors, enables reproducible treatments of a small volume (1 cm^3^ or less) with a precision of better than ±0.5 mm in stereotactic space. We hypothesized that this technology could be used to create a clinically relevant murine model of RN, using single hemispheric irradiation to generate late time-to-onset tissue injury whose histology matches that of patients with confirmed RN. An essential component of the critical evaluation of such animal models of disease and pathology is correlation of findings with the gold standard, histology. Previously, we have shown that late-onset tissue injury in mice irradiated with either the microRT system [9] or the GK [10–13] recapitulates the histologic features seen in patients with confirmed radiation necrosis. Herein, we report on the development of a semi-quantitative histologic *scoring system* for evaluation of the extent and severity of tissue injury in our GK mouse model of RN. Using this model, we demonstrate histologically the progression of injury following irradiation and establish a strong correlation between MRI-derived necrotic volume and a standardized histologic “score”. This validates both the mouse RN model and the use of MRI-derived indices for noninvasive, longitudinal quantification of the development and progression of RN in this model.

Overall, the goals of this work are to develop a GK-based murine model of radiation necrosis, to characterize the development of RN using serial MRI protocols and histologic evaluation, and to elucidate the critical determinants (i.e., radiation dose and fractionation scheme) governing the onset and progression of tissue injury. The well-characterized animal model of RN described and histologically validated herein provides a platform for mechanism of action studies, the identification of imaging biomarkers of RN, and the development of schemes for improved mitigation and neuro-protection.

## Materials and Methods

### Ethics Statement

All studies were performed in accordance with the guidelines of the Washington University Institutional Animal Care and Use Committee (Approval #20120030) with protocols that met or exceeded American Association for the Accreditation of Laboratory Animal Care standards. All irradiation and imaging experiments were performed under anesthesia, and all efforts were made to minimize suffering.

### Small-Animal Irradiation

**Fig 1:**
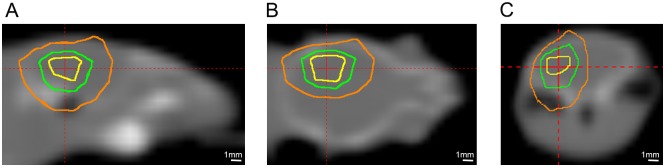
Gamma-Knife dose radiation contours superimposed on a CT image of a mouse. Panels A, B, and C show sagittal, coronal, and transaxial views, respectively. The three contours shown are at 75% (yellow), 50% (green), and 25% (orange) isodose. The red cross-hairs in each panel identify the location of the imaging planes shown in the other two panels.

Six-to-eight week old, female Balb/c mice (Harlan; Indianapolis, IN) were used for the study and observed daily and weighed weekly to ensure that interventions were well tolerated. Mice were irradiated with the Leksell Gamma Knife^®^ (GK) Perfexion™ (Elekta AB; Stockholm, Sweden; http://www.elekta.com/). They were supported on a specially designed platform mounted to the stereotactic frame that attaches to the treatment couch of the GK. Mice were anesthetized with a mixture of ketamine (25 mg/kg), acepromazine (5 mg/kg) and xylazine (5 mg/kg), injected intraperitoneally 5 min before the start of irradiation. Fig 1 shows Gamma Knife radiation isodose contours modeled using the GammaPlan^®^ treatment planning system and superimposed on the CT image of a mouse on the platform. The isodoses result from an irradiation using the 4 mm collimator aperture of the GK with all 192 sources focused on a single stereotactic position, whose location depends on the position of the mouse on the platform. This dose distribution illustrates that radiation is effectively concentrated to a single hemisphere of the mouse brain, and is greatly reduced in other radiation-sensitive anatomy, including the eyes or esophagus.

The immediate goal of this study was to devise an irradiation scheme that would provide an “experimentally tractable” (i.e., experimentally convenient) mouse model of late-onset RN. It was important that the irradiation scheme limit primary injury to the single ipsilateral hemisphere, that gold-standard histological measures of injury remain negative (absent) in the weeks immediately following irradiation, and that the late-onset appearance of RN occur within one to three months following irradiation. The focus on experimental tractability also motivated avoidance of highly fractionated (time consuming) irradiation schemes and a very late (many months) delayed occurrence of RN. Four cohorts of female Balb/c mice (n = 15 each) were irradiated with four different dose schedules: 60-Gy (50% isodose) in a single fraction, 60-Gy in three fractions given every 2 days, 50-Gy in a single fraction, and 45-Gy in a single fraction, as described below. Serial MRI examinations and histology were used to characterize the resultant brain parenchymal changes for each cohort. As will be shown, the 45- and 50-Gy single fraction irradiation schemes fully met the goal of providing an experimentally tractable mouse model of late-onset RN.

### MR Imaging

Images were collected in an Agilent/Varian (Santa Clara, CA) 4.7-T small-animal MR scanner equipped with a DirectDrive^TM^ console. The scanner is built around an Oxford Instruments (Tubney Woods, Abingdon, Oxfordshire, UK) 40-cm, clear-bore magnet equipped with 21-cm inner diameter, actively shielded Agilent/Magnex gradient coils (maximum gradient, 28 G/cm; rise time approximately 200 μs) and International Electric Company (IEC; Helsinki, Finland) model A–240 amplifiers (± 300 V and 300 A).

MRI data were collected using an actively decoupled coil pair: 1.5-cm outer diameter surface coil (receive) and a 9-cm inner diameter Helmholtz coil (transmit). Before the imaging experiments, mice were anesthetized with isoflurane/O_2_ (3% [vol/vol]) and maintained on isoflurane/O_2_ (1% [vol/vol]) throughout the experiments. Mice were restrained in a laboratory-constructed Teflon head holder with ear bars and a tooth bar. To maintain their body temperature at approximately 37°, mice were placed on a pad through which circulated warm water. Mice were injected intraperitoneally with 0.5 mL of MultiHance (gadobenate dimeglumine; Bracco Diagnostics) contrast agent, diluted 1:10 in sterile saline, immediately prior to positioning in the magnet.

Mice that received 60-Gy of radiation, in either single or three fractions, or single-fraction 50-Gy of radiation were imaged 4, 8, and 13 weeks post-irradiation. A subset of the mice that received single-fraction 45-Gy of radiation were imaged 4 weeks post-irradiation (no effect observed), and all mice making up the cohort were imaged at 7, 10, 13, 16, 19 and 22 weeks post-irradiation. The time interval between adjacent imaging sessions was chosen to ensure observable progression of RN on anatomic MR images. Mice were sacrificed immediately after the last imaging point (13 and 22 weeks post-irradiation for mice receiving 50/60-Gy and 45-Gy of radiation, respectively). Multislice, T2-weighted, spin-echo transaxial images were collected beginning ~3 minutes following intraperitoneal injection of contrast agent, with the following parameters: repetition time (TR) = 1.5 s, echo time (TE) = 0.05 s, field of view (FOV) = 1.5 x 1.5 cm^2^, slice thickness = 0.5 mm, 21 slices; total acquisition time = 12 minutes. Multislice, T1-weighted, spin-echo transaxial images were then collected over five minutes with the following parameters: TR = 0.65 s, TE = 0.02 s, with the same geometry as the T2-weighted images.

### Measuring Necrotic Volumes Quantitatively by MR Imaging

Regions of radiation necrosis appeared hyperintense on both contrast-enhanced T1-weighted and T2-weighted images. While either set of images could be used for quantitative analysis, we chose to calculate necrotic volumes from T2-weighted images, using image intensity histograms, as described previously [11].

### Histology

Selected groups of the mice were sacrificed for histological studies at various times after irradiation. Following intra-cardiac perfusion with 0.1 M phosphate-buffered saline and formalin, the brain was exposed *in situ* by removing the calvarium, and fixation continued by immersing the head of each mouse in formalin for 24 hours. The brain was then extracted from the skull, and coronal sections were prepared, processed through graded alcohols, embedded in paraffin, and sectioned. Eight-micron-thick tissue sections were stained with hematoxylin and eosin (H&E). Briefly, the slides were de-paraffinized with 100% xylene, 100% ethanol, 90% ethanol, 80% ethanol, and finally distilled water. Nuclei were stained with the alum hematoxylin and rinsed with running tap water, differentiated with 0.3% acid alcohol, and rinsed again with tap water. Slides were stained with eosin for two minutes, followed by a series of dehydration steps. Other samples were fixed in modified Zenker’s solution and stained, according to standard protocols, with either phosphotungstic acid-hematoxylin (PTAH) to demonstrate the presence of fibrin in tissue or with trichrome to reveal collagen deposition within the brain.

### Statistical Analysis

The differences in MR-derived necrotic volumes for mice having different histological scores were summarized using means and standard deviations, and compared by one-way ANOVA. Due to the relatively large variability in MR-derived necrotic volumes, a logarithm transformation was performed to better satisfy the assumption of a normal distribution [14]. The correlation between MR results and histology scores was assessed using Kendall’s tau correlation coefficient [15]. All the tests were two-sided and a p-value of 0.05 or less was taken to indicate statistical significance. Statistical analysis was performed using SAS 9.3 (SAS Institutes, Cary, NC). The congruence in histologic scoring among multiple raters was assessed using the SAS macro MAGREE (http://support.sas.com/kb/25/006.html) and the strength of agreement was described by Kendall's coefficient of concordance, which ranges from 0 (no agreement) to 1 (complete agreement).

## Results

### MRI detects radiation necrosis as image hyperintensity in T2-weighted images

**Fig 2:**
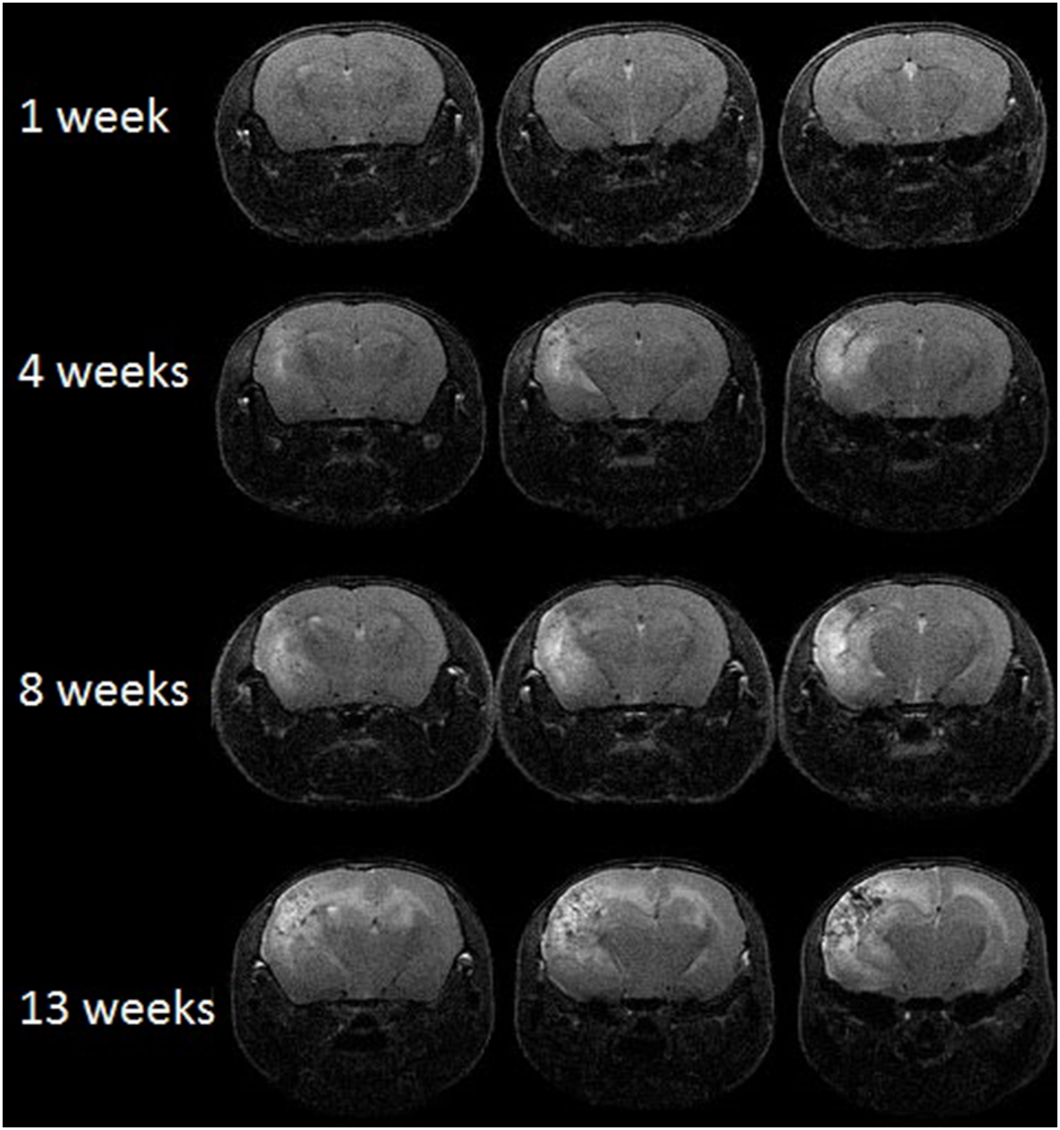
MRI can monitor radiation necrosis in irradiated mouse brain. Representative contrast-enhanced, transaxial T2-weighted spin-echo images of irradiated mice at 1, 4, 8, and 13 weeks following a single 50-Gy (50% isodose) of radiation. Slices are chosen to display the same anatomic region of the brain at all four time points.

Representative contrast-enhanced T2-weighted spin-echo images of irradiated mice, covering the same anatomic region of the brain and collected at 1, 4, 8, and 13 weeks following a single 50-Gy dose of radiation, are shown in Fig 2. Though not included in the volume measurements, some mice were imaged as early as one day post-irradiation, with no lesion observed. Hyperintense areas in these images, due to edema, correspond with regions of radiation necrosis in the brain. MR images begin to show hyperintense regions at ~4 weeks post irradiation, and these regions expand significantly in extent by 13 weeks, indicating late onset and rapid progression of radiation necrosis.

### Radiation dose schedules affect the onset and progression of radiation necrosis

**Fig 3:**
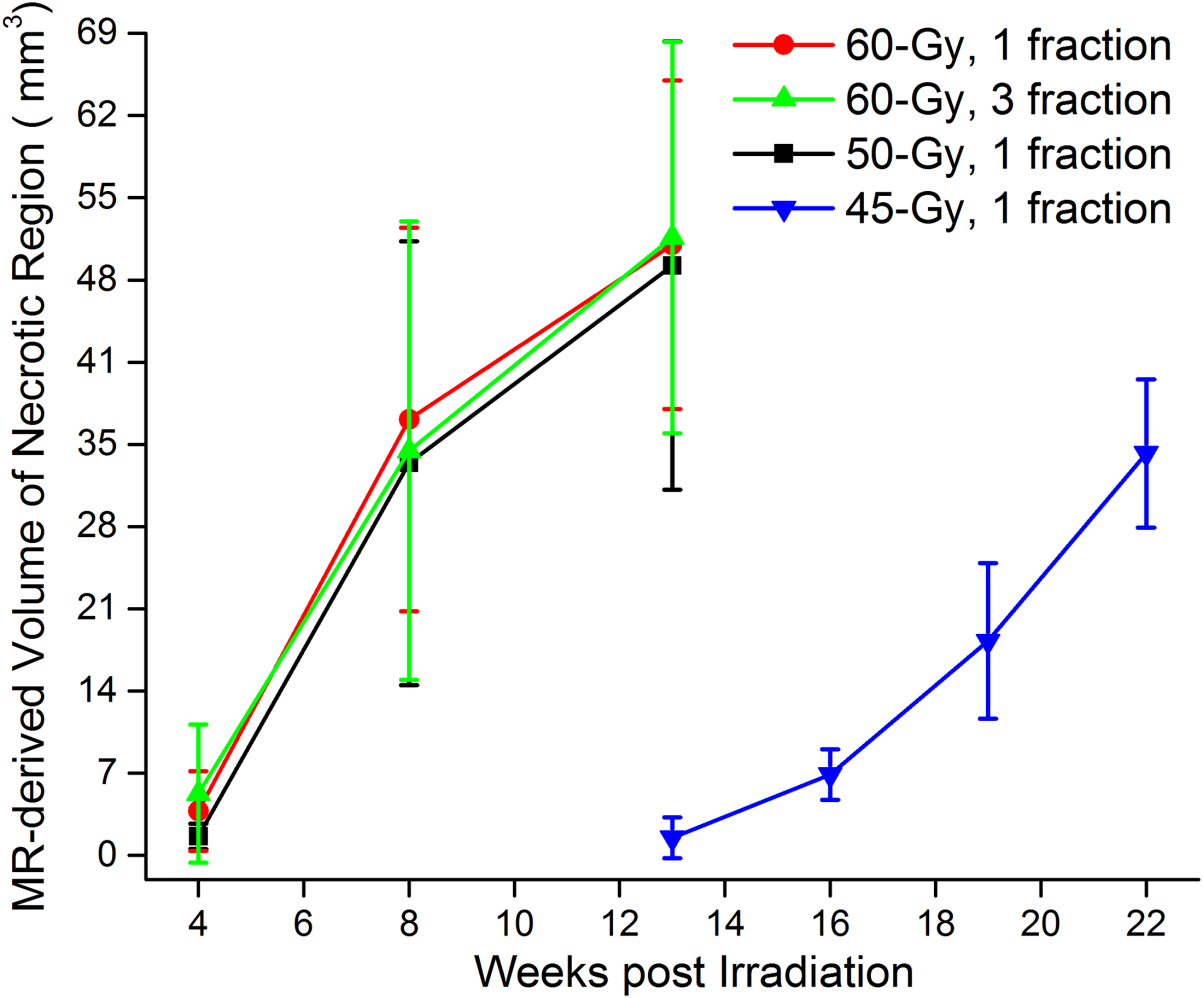
MRI-derived necrotic volumes. Mean volumes ± SD (n = 15), *vs*. time post-irradiation for mice irradiated hemispherically with different dose schedules: 60-Gy in 1 fraction (red), 60-Gy in 3 fractions (green), 50-Gy in 1 fraction (black) and 45-Gy in 1 fraction (blue).

Fig 3 shows the progressions of the volumes of MRI-derived necrotic regions for irradiated mice, in which mice received four different radiation dose schedules, including 60-Gy in a single fraction (red), 60-Gy in three fractions (green), 50-Gy in a single fraction (black), 45-Gy in a single fraction (blue). Mice that received either 60-Gy or 50-Gy of radiation developed RN 4 weeks post irradiation and had significant, expansive RN (>30% of the volume of the irradiated hemisphere), as determined by anatomic MRI, at approximately 13 weeks post irradiation. While time-to-onset of necrosis following 45-Gy irradiation delivered in a single fraction occurs much later than with 60-/50-Gy irradiation, the pattern of injury progression following onset is similar.

### Neuropathological grading system in the murine model of radiation necrosis

**Fig 4:**
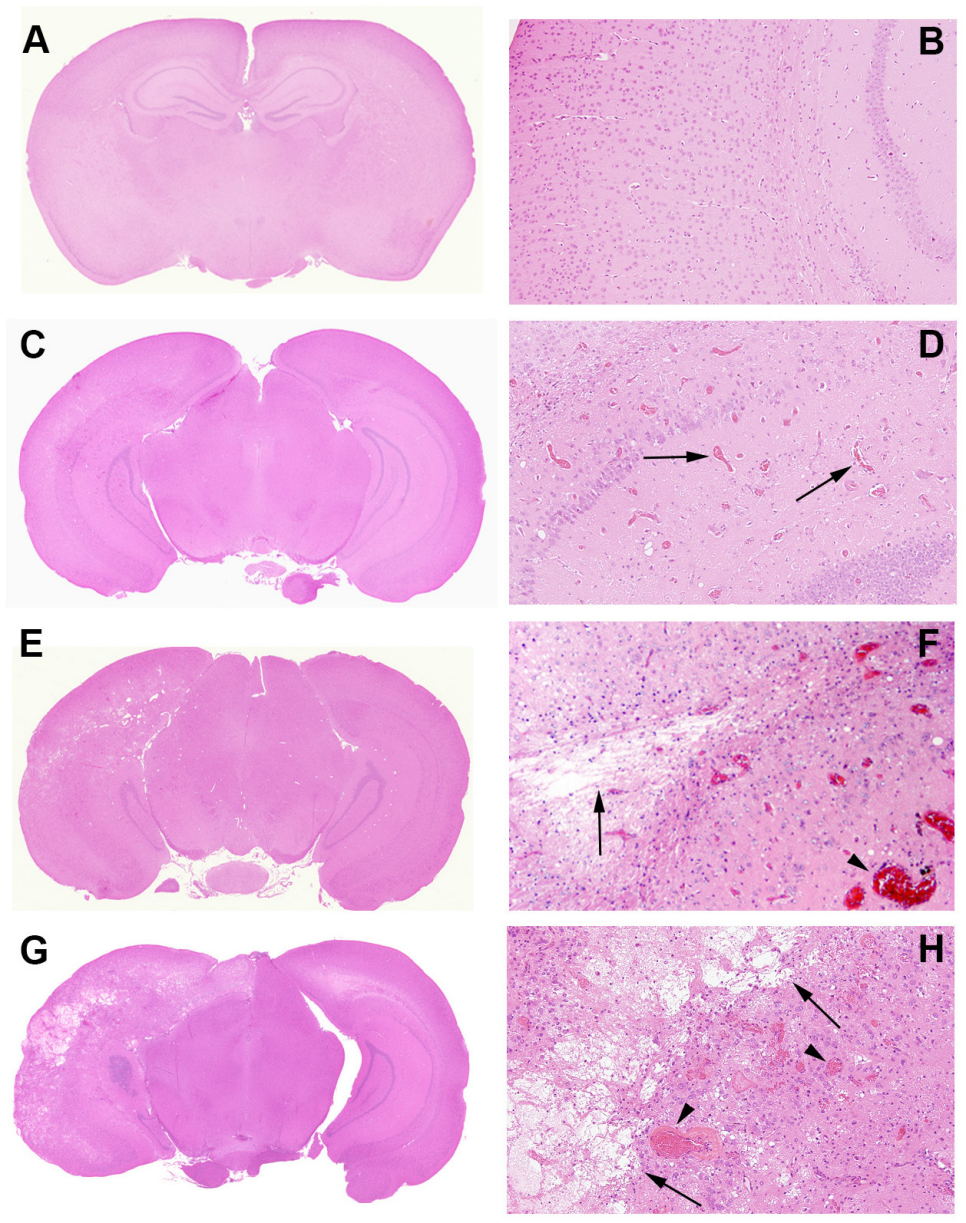
Representative histologic slides showing irradiation damage of each grade. All mice were irradiated on the left hemisphere, as displayed. Panels A, C, E, and G are shown at 1x; Panels B, D, F, and H are shown at 10x magnification, thereby revealing more detail in the irradiated areas. **Panels A and B (Grade 0):** The irradiated brain shows no pathological changes. **Panels C and D (Grade 1):** The earliest histological events were micro-hemorrhage accompanied by increased numbers of telangiectatic vessels (arrows). **Panels E and F (Grade 2):** Typically larger parenchymal hemorrhages (arrowhead) and tissue loss (arrows) are observed, with approximately one-half to two-thirds of the left hemisphere involved. **(Panels G and H (Grade 3):** More extensive areas are typically involved, with severe tissue damage, including fibrinoid vascular necrosis (arrowheads) and tissue loss (arrows).

**Fig 5:**
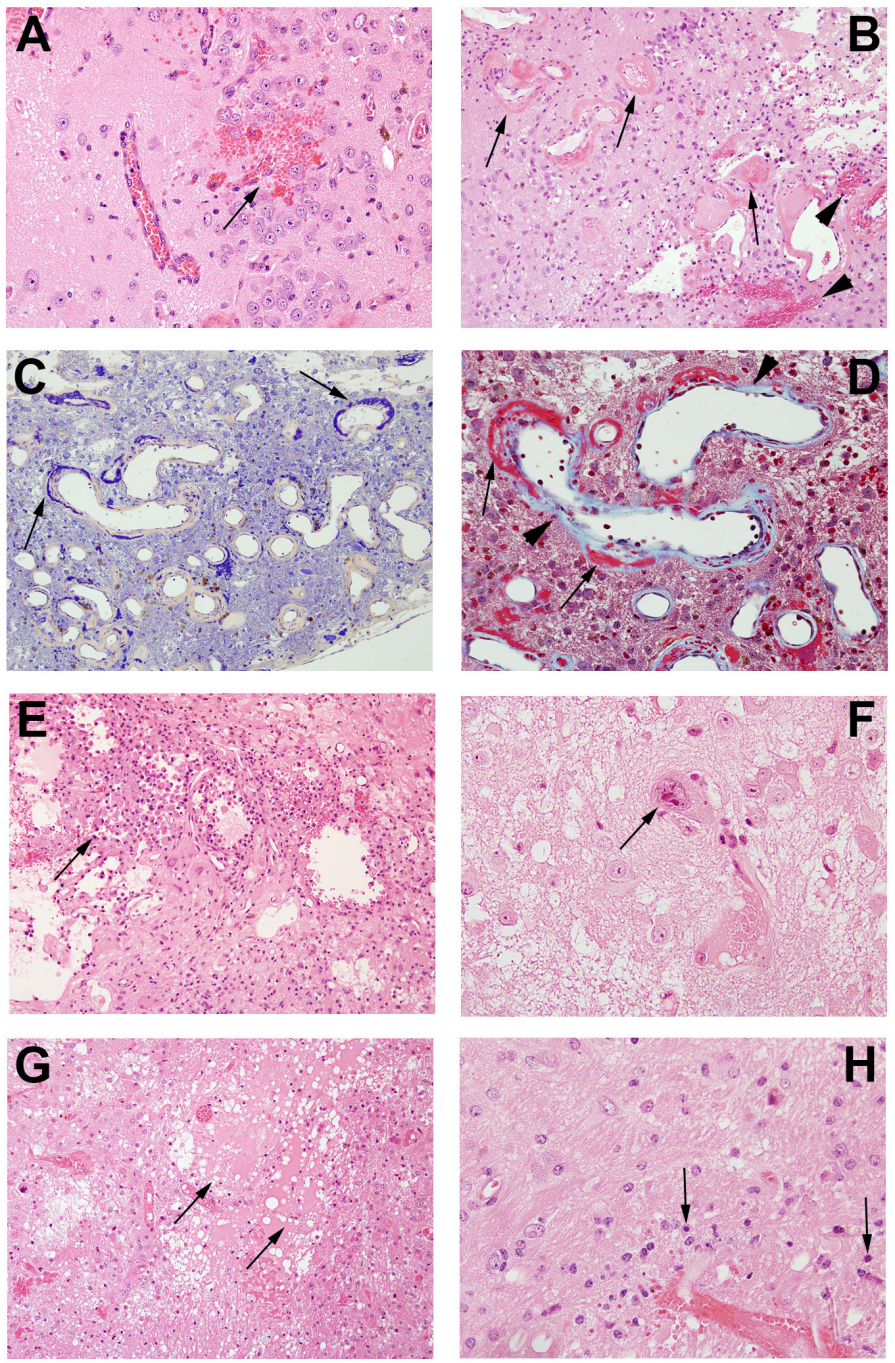
Pathological features in post-irradiation mouse brain. **A.** Micro-hemorrhage and dilated vessels (arrow, 20X); **B.** Hemorrhage (arrowheads) and fibrinoid vascular necrosis (arrows) (H&E staining, 20X); **C.** PTAH staining shows fibrinoid vascular necrosis in dark blue (arrows, 20X); **D.** Trichrome staining demonstrates fibrinoid vascular necrosis (red, arrows) and collagen deposition (light blue, arrow heads)(60X); **E.** Macrophages surrounding damaged tissues (arrow, 20x); **F.** Cellular atypia (arrows_,_ 60X); **G.** Edema (arrows_,_ 20X); **H.** Neuronal necrosis (arrows, 60X).

Histologic changes are shown in control and irradiated mice (Figs 4 and 5). Changes include microhemorrhages, edema, vascular changes ranging from increased numbers of delicate telangiectatic vessels to fibrinoid vascular necrosis and hyalinization, development of foamy macrophages, infiltration of polymorphonuclear leukocytes, astrocytosis, and loss of tissue elements, ranging from neuronal loss to frank involvement of all tissue elements with microcavitation. The severity of histologic changes for a total of 96 mice was independently scored by three individuals, including a clinical neuropathologist (RES), in blinded fashion, using a semi-quantitative, 0 to 3 grading system. Fig 4 shows typical changes characteristic of radiation necrosis in the weeks following irradiation of the murine brains at both low and high magnifications. *Grade 0* was assigned to no histologic changes (Fig 4A and 4B). *Grade 1* described mild histological changes associated with radiation injury, including early events such as micro-hemorrhages accompanied by increased numbers of telangiectatic vessels (Fig 4C and 4D). *Grade 2* injuries (Fig 4E and 4F), were characterized by moderate histologic changes, including changes seen in Grade 1 with the addition of vascular hyalinization, astrocytosis, and tissue loss. *Grade 3* injuries (Fig 4G and 4H) resembled those of Grade 2 but were more severe, often including extensive tissue loss. Grade 3 injuries also more frequently featured fibrinoid vascular necrosis. The agreement in histologic grading amongst the three independent scorers was measured *via* Kendall’s concordance coefficient. The calculated value of Kendall’s coefficient was 0.86 (p<0.0001), indicating substantial concordance in grading amongst the scorers.

A more detailed characterization of Grade 3 lesions (Fig 4G and 4H) is shown in Fig 5. Micro-hemorrhage and dilated vessels (Fig 5A) were observed in a majority of irradiated animals. The most severely affected animals showed hyalinized vessels and fibrinoid vascular necrosis (Fig 5B); extensive fibrin deposition, highlighted by PTAH staining (blue regions; Fig 5C); and trichrome staining (red-stained regions; Fig 5D). Collagen surrounding the vessel walls was also visualized with trichrome staining (blue material, Fig 5D). These vascular changes were accompanied by macrophages clustered in damaged areas of necrosis (Fig 5E) and a few cells that showed radiation-induced atypia (Fig 5F). Edema formed in the center of the damaged regions of brains (Fig 5G), with decreased neuronal/glial cell density and a paucity of blood vessels and parenchymal structures. Degenerating neurons were characterized by loss of cytoplasm and condensed pyknotic nuclei (Fig 5H).

### Histological scores for radiation necrosis correlate with MR-derived necrotic volumes

**Fig 6:**
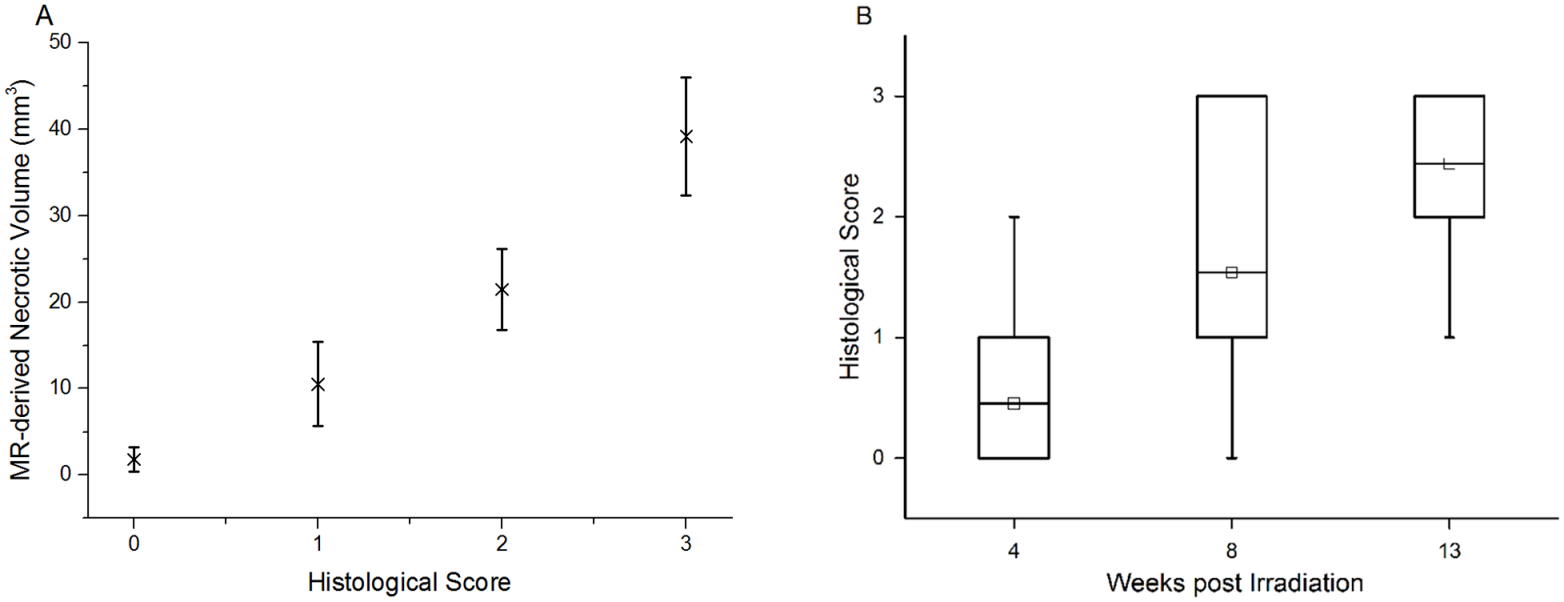
Histologic scores correlate with MR-derived necrotic volumes and post-irradiation time. **A.** Correlation between MR-derived necrotic volumes and histological scores. MR-derived volumes, mean ± SD (n = 30, 37, 27, and 53 for Grade 0, 1, 2, and 3, respectively), of radiation necrosis *vs*. histological score for irradiated mice, independent of specific radiation dose (45–60 Gy) and treatment schedules. **B.** Correlation between histological scores and time post irradiation. Box-and-whisker plots of the histological scores for mice receiving 50/60-Gy of radiation, at 4, 8, and 13 weeks post irradiation. The 25th-75th percentiles are blocked by the box, the band inside the box is the mean, and the whiskers mark the maximum and minimum values.

Consistent with the earliest appearance of hyperintensity in MR images, tissue damage was first noted at three to four weeks post radiation in animals that received a single fraction of 50- or 60-Gy of radiation. By histologic examination, no specific changes suggestive of radiation injury were observed at earlier post-irradiation time points. For each graded histology slide, the MR-derived necrotic volume was calculated from the T2-weighted image for a single slice, covering the same anatomic features as the histological slide. The average MR-derived necrotic volumes *vs*. histological scores are plotted in Fig 6A. The two measures are highly correlated (correlation coefficient = 0.948, with p<0.0001) and the differences among the four cohorts (n = 30, 37, 27, and 53 for Grade 0, 1, 2, and 3, respectively) are all statistically significant (p<0.0001). Further, the correlation between histological score and time post irradiation (correlation coefficient = 0.508, with p<0.0001) is displayed in Fig 6B. The differences among the three cohorts (n = 17, 15, and 20 for 4, 8, and 13 weeks following 50/60-Gy of radiation) are all statistically significant (p<0.0001). These strong correlations help to validate the MR results and demonstrate that the histologic grading system is a useful tool for the classification of the severity of radiation necrosis.

## Discussion

An optimized mouse model of cerebral radiation necrosis incorporates several important features, including: i) consistent induction of late time-to-onset necrosis following irradiation; ii) characteristic MR imaging changes that allow clear identification of necrotic regions; iii) tissue injury whose histology accurately matches pathological findings in brain tissue from patients with confirmed radiation necrosis; iv) progression of necrosis occurs over an experimentally appropriate period of time, thereby enabling longitudinal imaging studies to characterize the onset and development of necrosis and its response to therapeutic interventions. Herein, the initiation and progression of necrosis in irradiated mice subjected to different radiation doses and fractionation schedules (60/50/45-Gy in a single fraction and 60-Gy in three fractions) were compared. We evaluated higher RT doses in the mouse model then those normally clinically administered to ensure the development of late cerebral RN in all cases. Our results demonstrate that mice receiving 60 Gy and mice receiving 50 Gy have similar patterns of RN onset and similar patterns of RN progression. Time-to-onset of necrosis following a single-fraction of 45-Gy irradiation occurs much later than with higher doses. Fractionation did not significantly delay onset of late cerebral RN compared to a single fraction, presumably due to the relatively high total RT doses (45–60 Gy) involved. In all cases, the onset of injury observable by either MRI or histology is delayed by several weeks following the irradiation. Thus, this mouse model truly illustrates late-onset injury, which is a hallmark of radiation necrosis.

The radiation dose-response relationship can be represented graphically by a curve that maps the biologic effects observed in relation to the radiation dose received. A variety of different linear and non-linear models, some including a threshold radiation dose for initiating tissue damage, have been proposed [16]. The sigmoid threshold curve of radiation dose-response is commonly employed in radiotherapy to demonstrate high-dose cellular response. Changes in the induction threshold for radiation injury have been described as shifts toward the right or left of this sigmoid curve [16]. In terms of the development of RN, a decrease of radiation dose from 50-Gy to 45-Gy causes a much more dramatic difference than a decrease from 60-Gy to 50-Gy, suggesting that the rapidly changing part of the sigmoid curve includes the 45–50 Gy range (Fig 3).

Consistent with this proposition, we have successfully employed radiation doses of 45 Gy in studies with GSK–3β inhibitors as neuroprotectants [11] and 50 Gy in studies of anti-VEGF antibodies as mitigators of RN [10]. These radiation doses provide appropriate observation windows (from 4-to–13 weeks post irradiation) for the development of late-onset necrosis, with evident weekly changes in the volume of the necrotic region. While these radiation doses are somewhat higher than standard clinical doses in single-fraction, stereotactic radiosurgery (typical radiation doses for treatment of brain tumors or arteriovenous malformations [17] are 15 Gy and 24 Gy (50% isodose), respectively), our goal was to induce RN in all cases, rather than avoid or minimize its severity. Radiation necrosis is difficult to distinguish from recurrent tumor using conventional, anatomic, T1- and T2-weighted MRI. We, and others, have explored the use of advanced MR methods, such as diffusion-weighted [DWI], magnetization transfer [MT], dynamic contrast enhanced [DCE] (vascular permeability) and dynamic susceptibility contrast [DSC] (perfusion) MRI methods for distinguishing RN and tumor [12,18–24]. The observation of RN that is histologically similar to that induced in patients suggests that our mouse model can serve as a platform for the identification and validation of imaging biomarkers, and can be readily translated to support the development of improved clinical treatment paradigms.

## Conclusions

A novel, robust murine model of late time-to-onset radiation necrosis that recapitulates significant features of RN that occur in response to a number of clinical treatment paradigms, including the late onset of radiation changes and well-described histologic changes, has been developed. A semi-quantitative histologic grading system was developed to assess the severity of the radiation-induced changes. Changes in necrotic volumes measured by MRI correlated well with histologic score derived based on this grading system. This mouse model of RN can, thus, serve as a robust platform for a variety of studies to test the efficacy of various treatment paradigms, examine mechanisms involved in the onset and progression of delayed RN and its response to therapeutic interventions, and develop imaging biomarkers for differentiating RN from recurrent tumor.

## Supporting Information

S1 Table Animal Research: Reporting In Vivo Experiments (ARRIVE) Guidelines. Completed ARRIVE checklist.
(PDF)
